# Linking public leadership with project management effectiveness: Mediating role of goal clarity and moderating role of top management support

**DOI:** 10.1016/j.heliyon.2023.e15543

**Published:** 2023-04-20

**Authors:** Muhammad Zada, Jawad Khan, Imran Saeed, Shagufta Zada, Zhang Yong Jun

**Affiliations:** a Business School Henan University, Kaifeng, 475000, People's Republic of China; b Facultad de Administración y Negocios, Universidad Autónoma de Chile, Santiago, 8320000, Chile; c Department of Business Administration, Iqra National University, Peshawar, Pakistan; d Institute of Business & Management Sciences (IBMS), The University of Agriculture Peshawar, Pakistan; e Department of Business Administration, Faculty of Management Sciences, Ilma University, Karachi, Pakistan

**Keywords:** Public leadership, Goal clarity, Top management support, Project management effectiveness

## Abstract

**Objective:**

Grounding on social learning theory (SLT), this study examines the effect of public leadership on project management effectiveness (PME). Further, this study examines the mediating role of goal clarity and moderating role of top management support.

**Methodology:**

Hierarchical linear regressions were used to investigate the relationships. PROCESS Hayes (2003) Model 7 was used for the moderation and mediation analysis. The data was collected from 322 Pakistani public sector developmental project employees.

**Findings:**

The results show that public leadership positively affects goal clarity (β = 0.049, p < 0.001) and project management effectiveness (0.032, p < 0.001). In addition, goal clarity mediates the association between public leadership and project management effectiveness (0.36, p < 0.001). Furthermore, the strength of the mediated relationship between public leadership and project management effectiveness (via goal clarity) depends on top management support. The indirect effect of public leadership on project management effectiveness is high when top management support is high (compared to low).

**Conclusion:**

The role of public leadership contributes significantly to the project's success. The project leader recognises, enlists, and promotes the organisation's core competencies, identifies, corrects, and controls key rigidities, places a high value on goal clarity, and continually lines up procedures with the project's overarching goals.

**Implications:**

Public leadership is crucial in project management effectiveness, especially in the public sector, where projects often involve multiple stakeholders, limited resources, and complex regulatory requirements. Effective public leadership ensures that projects are aligned with the organization's mission and goals and carried out efficiently, on time, and within budget.

## 1 Introduction

Governments worldwide use national development plans and public sector projects for socio-economic development. Pakistan's government and funding agencies consume a considerable share of the national budget on public sector developmental projects [1]. The leadership envisions the public projects formulated with input from the central planning commission and provincial planning departments. Various governmental agencies are responsible for executing the projects. Public project officials located across the country supervise the agencies during project implementation. The projects' ultimate goal is to serve the socio-economic interests of the general public [2]. Project management effectiveness (PME) aims to measure, develop, and proficiently accomplish the project from initiation to execution and implementation [3]. Project management effectiveness also includes determining the project's scope, developing the procurement procedures, keeping track of progress, and making payments to the treasury office within a set time frame.

However, public management institutions and scholars recently interpreted project management effectiveness differently because of projects' changing nature and purpose. The British Association of Project Management (BAPM) and Project Management Institute (PMI) measure public project management effectiveness against the criteria of meeting triple constraints and stakeholders' satisfaction [4]. Management scholars suggest measuring public project management effectiveness against multidimensional criteria, including meeting project management effectiveness, generating organisational benefits, providing certain socio-economic assistance, future potential, and stakeholders' satisfaction [5].

Project managers oversee all aspects of a project, including technical issues. Yet, there is still a prevalence of scientific studies that suggest project managers tend to focus more on project goal issues rather than technical problems that can function as significant bottlenecks to project management effectiveness. Indeed, the leadership competencies of project managers highly influence project management effectiveness [6]. Podsakoff [7] asserted that the significance of project managers' human interactions and leadership styles (i.e., public leadership) are vital components of project management effectiveness. Project management and public leadership are critical components of successful public sector organisations. Effective project management involves planning, leading, organising, mentoring and controlling resources to achieve specific goals within a defined timeframe. Public leadership, on the other hand, involves guiding and motivating individuals and groups to work towards a common vision or goal [3]. The effectiveness of project management and public leadership is closely linked, as strong leadership is essential for effective project management. A public leader with the skills and knowledge to manage projects effectively can help ensure that projects are completed on time, within budget, and to stakeholders' satisfaction. This requires technical project management skills and the ability to communicate effectively, minimise project failure, manage conflict, and motivate teams toward organizational effectiveness.

**Fig. 1:**
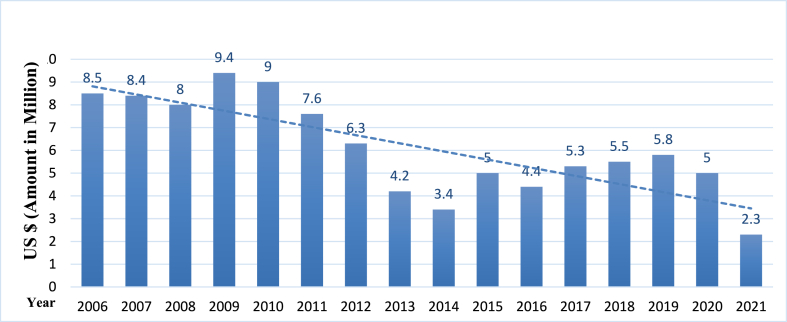
Source: ADB (2021).

Project failure can significantly challenge public sector organisations and their leaders. When a project fails, it can result in financial losses, damage to reputation, and negatively impact public services. Therefore, public leaders need to understand how to handle failure effectively and minimise its impact on the organisation and its stakeholders. As per the published report, Pakistan paid about a $100 million fine to the Asian Development Bank (ADB) for failing to carry out a specific number of public projects over the previous 15 year [8]. Implementing the project leader's role is important. In Pakistan, because of poor leadership in implementing donor-funded projects for which the valuable hard-earned foreign exchange reserves of about $100 million were paid by different governments taking office since 2006 (see Fig. 1). So, public leadership plays an important role in streamlining public projects to define goals clearly and works on project efficiency.

### 1.1 Sovereign and non-sovereign loans penalty

Leadership is an influential factor of project management that engages the followers and guarantees they are more aware of and connected with whatever is happening, allowing for more accurate judgments [9]. According to Zulkiffli and Latiffi [10], 80% of project failures are because of poor leadership. Project failure and success both highly rely on the leadership approach of project managers [11]. Project managers are held accountable for the success or failure of their projects. As the individual overseeing the project, the project manager is expected to ensure that it is completed on time, within budget, and to the satisfaction of stakeholders [10].

Pakistan's public sector developmental projects have played a critical role in the country's economic and social development over the years. These projects cover a wide range of sectors, including infrastructure, education, healthcare, energy, and agriculture, and are designed to address some of the country's most pressing challenges [4]. Public leadership plays a critical role in the success of these projects. Effective public leaders are responsible for guiding and motivating their teams, setting clear goals and objectives, and managing resources to ensure that projects are completed on time, within budget, and to the satisfaction of stakeholders [5]. However, the public sector in Pakistan faces a range of challenges that can impact the effectiveness of public leadership in delivering successful developmental projects. These challenges include corruption, political instability, and bureaucratic inefficiencies [12].

According to Tummers and Knies [13], public leadership encourages team members to be transparent and honest about their and their organisation's actions and ensure their team members comply with laws, rules, and procedures. According to the social learning theory (SLT), people learn new abilities by seeing how those around them behave (models) [14]. The authors assess the effect of such actions by observing the good and bad effects after engaging in them. There are several ways in which social learning theory may be used as a framework for understanding leadership, including how it can be used to better understand a leader's behaviour and how it can be used to recognize the context in which the leader operates. According to SLT, employees actively learn leadership behaviours through vicarious learning or role modelling [15]. Employees cognitively evaluate and process information about their leadership and, consequently, adopt attitudes and behaviors expected to be admired by the leadership [16]. These behavioral learning processes work efficiently in leader-follower relationships because of the job position and status held by the managerial leadership [17].

Social learning theory supports that public leadership style influences project effectiveness by altering attitudes and goal clarity [18]. To follow the rules, action plans, and procedures formulated in advance for executing projects, to remain loyal and committed to the execution of the projects, and encouraged and motivated to work together and develop networks with the internal and external stakeholders of different projects, ultimately lead to project management effectiveness [19].

Goal clarity is an essential concept of project-based research and has widespread theoretical and practical implications across various disciplines, including project management [20]. It is widely studied across various disciplines; however, insufficient research regarding teams in the organisational effectiveness context [13]. The present study is aimed to fill this theoretical void by proposing a dynamic indirect effect model in which public leadership impacts project management effectiveness via goal clarity. Goal clarity reflects leaders' professional and objective-oriented behavior on subordinates' professional attitude, such as the concept of a Canndo approach, because the latter includes its competence and perceived impact at work [20]. Goal clarity is considered one of the most central traits of public project managers and an essential precursor of project management effectiveness [21].

Successful and productive team goals, coordination, coherence, and participation are vital to goal clarity and influence project management effectiveness [22]. We employ theories of motivational spirals on how leadership is pursuing goals by followers through the leader's direction and influence [23]. The sharing objective nature of leaders contributes to their desire to function together, and thus followers understand the job roles and project goals, which improves the overall project productivity [3]. Employees who feel motivated, independent, meaningful, and speak up should promote well, particularly in a project-based association. Vogel and Masal [24] asserted that public leaders embrace adherents' skills, ambitions, and feedback appreciation to execute the project excellently. The prior studies explored the different leadership styles to discover the usefulness of goal clarity in project management effectiveness [20]. Therefore, we follow the footprints of prior literature and propose that goal clarity mediates between public leadership and project management effectiveness.

We suggest that the perception of the top management's support be the first layer through which the public leader affects goal clarity. Professional public leaders cannot effectively execute team goals until the top management supports them. Research shows leadership behavior and top management support are essential for goal clarity [19]. Past studies demonstrated the need for top management support as a critical factor in the different project stages [25]. However, researchers scarcely discussed the reciprocal role of leadership and top management support in goal clarity [26]. Top management support is answerable for plan construction, and it must include relevant information and experience on the prevalent scenarios within organisations during the project periods.

**Fig. 2:**
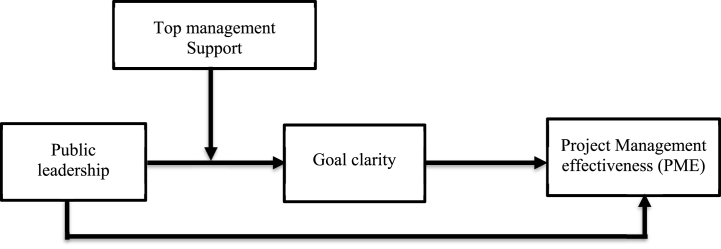
Theoretical model.

In light of the above arguments, the existing research proposes an empirical analysis of a hypothetical model that recommends the association between public leadership and PME with goal clarity as the arbitrating method, whereas the top management support moderates the outcomes. By examining how public leadership is connected to project management effectiveness in the arbitrating possessions of goal clarity and the sensible role of top management support, the present study aspires to add to the public leadership and SLT literature in numerous ways. First, this study investigates the connection between public leadership and project management effectiveness. This would provide a better understanding of the relationship between top-to-bottom leadership practices in different project-based organisations. Second, goal clarity plays crucial role in mediating the relationship between public leadership and project management effectiveness. Third, this study used top management support as the moderator, creating a favorable atmosphere and enabling a public leader to accomplish robust goal clarity and PME (see Fig. 2: a conceptual model).

## 2 Literature review and hypotheses development

### 2.1 Public leadership and project management effectiveness

Based on Social learning theory (SLT), public leadership can significantly impact project management's effectiveness. Effective public leaders can inspire their team members and stakeholders, promote accountability and transparency, and foster a culture of collaboration and innovation [27].

“People are social beings and want interaction, and social learning is the primary form of learning, just as word of mouth advertising is the highest form of advertising [28].”

According to SLT, individuals learn attitudes and behaviors more actively from their leaders through vicarious learning or role modeling [15,29]. The observation that people acquire new patterns of behaviour either via first-hand experience or by seeing the actions of leaders gave rise to the concept of social learning, which subsequently affected the organisation effectiveness. Social learning theory states that subordinates process and evaluate the leader's approach and adopt their attitudes and behaviors in a way expected to be admired by their leadership [29]. These attitudinal and behavioral learning processes work efficiently in leader-follower relationships because of the job position and status held by the leader [15]. In particular, public leaders who prioritise accountability can help ensure that project goals are achieved transparently and honestly. They can encourage their team members to be open about their actions and decisions and to communicate effectively with stakeholders. This can help to build trust and credibility with stakeholders and improve project outcomes [18].

Public leader's influences project management effectiveness by altering the attitudes and behaviors of team members in various manners: 1) public project managers encourage and emphasise showing open and honest sharing of personal and project unit actions and information to internal and external stakeholders. Actions and information sharing may influence stakeholders' participation and satisfaction with the project [30]. Project-related information sharing and stakeholders' participation and involvement are positively related to project management effectiveness in terms of efficiency, impact, and stakeholders' satisfaction [31]. Actions and information sharing may improve team members' expertise in correcting actions and procedures for executing projects. Sharing and following project actions and working methods are related to project efficiency [19].

2) Rule-following leadership: Public project managers emphasise properly following the law, rules, regulations, and procedures [32] and may alter team member's attitudes and behaviors in a way that they follow the planned project's actions and procedures, with expectations that such behavioral actions are admired by the public project managers, which leads towards efficient project completion. Public projects are planned by taking the guidelines of the stakeholders. Therefore, following approved procedures and guidelines may improve stakeholders' satisfaction. Following well-established lines of action and procedures may facilitate team members to achieve the designed project outcomes efficiently. It may minimise safety and environmental concerns because physical safety and protection of the environment are ensured during public project planning. Proper procedure following may improve resource mobilisation as planned. Researchers demonstrate that well-formulated clear project plans and procedures are related to project management effectiveness [3]. Project efficiency, impact, stakeholders' satisfaction, safety and environment (sustainable development) are dimensions of public project success [18].

Some studies discuss that employees' rule-following behaviors lead to the adoption of the reforms of process management for achieving efficiency and improved performance [33] and may streamline behaviors towards the achievement of project task performance [34]; 3) Political loyalty leadership: when public project managers encourage followers not to jeopardise relationships with political heads and to defend the choices of political heads. Using SLT, the project team starts defending political heads' project-related choices. Choices of political leadership are that the project team carries out the planned goal actions and activities to generate certain predetermined outputs and impacts. It is also the choice of political leadership to present the project to satisfy other stakeholders, including the general public, through the project, as this may give political leverage during a political change [35].

Project managers' political loyalty dimension transfer loyalty and commitment to the project team to carry out public projects. Earlier literature notes that employees' loyalty is related to their voice behavior on projects [36] and satisfaction in public agencies [37]. Employee loyalty promotes project performance Rahimpour [38,39]; 4) Network governance leadership: when public project managers encourage and motivate team members to develop new contacts and work with different internal and external contacts/networks of stakeholders. Using SLT, the team members may learn to develop new internal and external contacts and may motivate to work together with their departmental networks. Developing new contacts and working with networks may influence project effectiveness differently. This may develop the required team members' professionalism to process the projects efficiently to generate outcomes [40]. Networking may attract stakeholders' involvement and support by receiving different hard and soft resources required for successful project completion [3]. Earlier literature has noted timely resource allocation and stakeholders’ involvement contribute to project success [5].

The existing empirical research has related the mainstream transformation leadership constructs to project success [18]. Vogel, Reuber [32] show that public leadership in the form of accountability, rule-following, political loyalty and network governance should follow a specific approach. The existing studies have found empirical effects of individual dimensions and the combined construct of public leadership on different public sector outcomes, including civil servants' commitment, engagement, motivation, service performance, citizenship behavior, change orientations [13], public service motivation, job performance [17], civil servants professionalism and public agencies effectiveness [40]. Grounding on SLT, this study assumes that the combined construct of public leadership is related to public project management effectiveness. Therefore, it is hypothesised that:

Hypothesis 1 Public leadership is significantly and positively associated with project management effectiveness.

### 2.2 The mediating role of goal clarity

Clear goals are essential for effective project management because they provide a framework for planning, organising, executing, and controlling a project. Project managers need to know what they are working towards, why it is important, and how success will be measured. This information helps them to develop a clear project plan, allocate resources appropriately, and keep the project on track [41]. The success standards will be exceptional if the goals are clear to the teammates [42]. If goals are not properly formulated, followers cannot be aware of the mission and intention of the project [21]. Explicit directions within the guidelines of their procedures permit the subordinates to self-manage their tasks, encouraging them to achieve organisation effectiveness [43]. The public sector project is also supposed to succeed with clear project goals; stakeholders' expectations are visible to the project manager and team members [44].

Addressing project goals to the team members is a critical component of leadership. The project aims and scope must be clear to the project team. Goal clarity and public leader's confidence lead to team stability and project management effectiveness [45]. The public leader articulates the objectives from the project's beginning and informs the project team members about their roles, plans, and goals accordingly [2]. Goal ambiguity or the lack of clarity about programmed parameters and purposes will lead to adverse results for project effectiveness [21]. Public leadership conduct would eradicate inconsistencies in project goals, conditions, and parameters to ensure the project is finished on set targets and schedule [46]. Public leaders often value subordinates' views and suggestions, increasing their confidence and enhancing competence [47]. Moreover, Public leaders' authority delegation and interactive behavior let workers feel liberated from hierarchical limitations. They have a dignity that may impact project management effectiveness.

Goal clarity is characterised as dedication, endurance, increased task performance, recognition of work roles, beliefs, norms, values, and standards that lead to project management effectiveness [48]. Employees become more knowledgeable, learn new techniques, and improve their capabilities, which are the key markers of project management effectiveness. The team works autonomously beyond the project management office and routine organisational structure. Autonomous, professional, adequate knowledge about project goals and task proficiency provokes team members to complete the project on a given timeline and approved budget [49]. A competent team promotes self-confidence in solving challenges creatively [45]. Subordinates are assured that their work efforts and contributions are enough to drive them to make additional efforts to execute the project effectively [43]. Past literature confirmed that team goal clarity has more effectively achieved set project targets [20]. Developing a positive concept of goals will lead to the outstanding and effective execution of the public project. As a result, the project will be carried out along with stakeholders' needs, desires, and satisfaction. The project's efficiency is viewed from the stakeholders' perspective, and if the stakeholder is pleased, the project will be recognised as efficacious [50]. Therefore, goal clarity would also contribute to a better view of the objectives that might lead to effective project execution.

Previous studies show that a public leader guide subordinates to carry out the project by inducing an effective goal clarity mechanism. Therefore, goal clarity is an organising principle to articulate public leadership's effect on project management effectiveness. Leaders set scope and objectives by providing adequate goal clarity through public communication. In turn, this information processing method motivates the follower to understand the job's goals and meaning, leading toward the effective execution of the project.

Hypothesis 2 Public leadership is positively and significantly related to goal clarity.

Hypothesis 3 Goal clarity mediates the relationship between public leadership and project management effectiveness.

### 2.3 The moderating role of top management support

Top management support is the extent to which workers think their employer supports and cares about them well enough and satisfies their social-economic interests. Top management support enhances employees' confidence, helps them formulate policies, standards, prudent goals, and provides directions and counseling for excellent management across the project [3]. Top management support encourages harmony and goal-setting [51]. Top management support is closely linked to public project managers and team members, such as team training and building, goal clarity, communication policies, and strategies [52]. Consequently, we assumed that the project managers' overview was inadequate to identify the project objectives or carry out the project efficiently without the top management's support.

Public sector supportive management delegate authority to project managers and consider their guidance, creating a collective impact on the workplace [53]. Such a cohesive and supportive organisational environment enhances public leadership and supporters' efficiency, essential for the project's management effectiveness [3]. A public project manager will only exercise the right to transfer power to others if the organisation assigns ample power. Call for target-orated feedback from project team members is an extra characteristic of a public project manager relevant to goal clarity and project management effectiveness. A public leader can only implement this consistency if the top management supports them, is less hierarchical, and is generous. This is an extension of prior studies, which have found that supportive management allows project management to be more effective and adoptive the project triumph pace [54].

Top management support is proficient in establishing an immediate training and team development culture [55]. As a public project leader, the impression of positive and supportive management allows the project manager to accept the weakness and mistakes toward ambiguous states [56]. Enable followers to sense mentally comfortable and share new thoughts on the solution to trials and errors [57]. The encouragement has been developed to increase team members' effectiveness with unique expertise to find flexibility and innovative solutions to workplace challenges [58]. Under top management support, any difficulties connected with the goal clarity and goal clarity process might be more readily addressed. However, any setback due to internal or external reasons will be quickly handled, pushing the project management effectiveness.

### 2.4 Integrated model

Social learning theory outlines the technique whereby individuals distinguish the quality of the information in their immediate goal-setting and team management connected to their attitudes and behaviors. Social learning theory discusses in depth how the social environment, for example, perceived top management support, in which individuals do their jobs, impacts behaviors and attitudes, implying that social interactions provide a lens through which individuals understand the meanings and goals of the project [59]. The work environment sustenance and protects the possessions of individuals, workers, and organisations and strengthens individual and group information transmissions. The perceived top management support influences leader and employee behaviors by creating meaning and bringing salient information to their attention [60]. Thus, top management support influences leaders' and employees' beliefs and understanding of set goals and objectives. Based on this reasoning, we argue that leaders' and employees’ perceptions of top management support make project goals clear. The display of top management support engenders the perception that public leaders and project team building strengthen project management effectiveness.

Hypothesis 4a A higher level of top management support moderates the association between public leadership on goal clarity.

Hypothesis 4b Top management support will moderate the relationship between public leadership and PME via goal clarity such that the mediating association is stronger at higher levels of top management than at lower levels.

## 3 Methodology

### 3.1 Population and sample

Data were collected from employees of public sector developmental projects in Pakistan through a questionnaire from June 2022 to October 2022. Informed consent was obtained from all participants. Leadership in the public sector is crucial because it influences the efficiency of the government and other public institutions and the productivity and satisfaction of public sector employees. We have approached the concerned department for data collection to get proper approval. The concerned persons of organisations have allowed us to conduct the surveys and directed the human resource management department to facilitate us in collecting data.

The data was collected through convenience sampling using a time lag study. We have maintained a gap of one month between phases to minimise common method bias. At the start of the data collection, we contacted 472 employees in phase 1 to collect data regarding demographics, public leadership, and top management support, and we received 436 questionnaires (92.4%). In phase 2, we contacted employees who participated in the first phase to collect data regarding goal clarity. We received 387 complete questionnaires in phase 2 (82.0%). During phase 3, only the respondents from the second phase were contacted, and we received 337 responses regarding project management effectiveness. Due to missing basic information, we excluded 15 questionnaires. Finally, a sample comprising 322 respondents was used for data analysis. The sample size comprised 21.0% females, whereas 79.0% were males. 8.2% of respondents were between 25 and 30, 55.4% were between the ages of 31 and 35, 21.5% were between 36 and 40, and 14.9% were older than 40.71.5% of the employees had a Master's degree, 16.8% had completed a bachelor's degree, and the remaining had Higher Secondary School Education (HSSC). One year of service was mandated as a minimum requirement for eligibility. Of the employees, 56.6% have one to five years of experience, while 3.3% have more than sixteen.

### 3.2 Measures

The constructs of the study were measured using standardised instruments adopted from earlier literature. Responses were received on a five-point Likert scale where “1″ stands for strongly disagree to “5″ for strongly agree.

**Public leadership:** Public leadership was measured using the shortest 11-item scale recommended by Ref. [32], adapted from the original scale of [13]. The Cronbach s alpha (α) reliability of the instrument was 0.93.

**Goal clarity:** Goal clarity was measured using a 3-items instrument developed by Hoegl and Parboteeah [61]. The Cronbach s alpha (α) reliability of the instrument was 0.78.

**Top management support (TMS):** Top management support was measured using the 6-item scale developed by Islam, Doshi [62]. Cronbach s alpha (α) reliability of the instrument was 0.93.

**Project Management effectiveness (PME):** Project Management effectiveness was measured with a 10-item items scale developed by Ong and Bahar [63]. Cronbach s alpha (α) reliability of the instrument was 0.92.

**Control Variable:** To test our hypotheses, we included gender, organizational tenure, and education as potential control variables because of their potential association with public leadership and its outcomes [18].

## 4 Results

### 4.1 Confirmatory factor analysis

We used AMOS to carry out several confirmatory factor analyses to verify the factor structure of our survey instruments [64]. After executing several CFA episodes, the five-factor model gave the best match: χ^2^/df = 2.56, χ^2^ = 171.62, p < 0.001, SRMR = 0.05, TLI = 0.90, CFI = 0.91, RMSEA = 0.07, GFI = 0.92, as shown in Table 1. We can test the study's main hypotheses after we have a proof for our hypothesised 5-factor model. Furthermore, when all the items were loaded onto a single common method factor, the resulting variance was 0.265, less than the 0.50 cutoff score [65]. This study's results demonstrated that the common method variance was insignificant.

**Table 1:** Confirmatory factor analysis.

Model's	GFI	RMSEA	CFI	TLI	SRMR	X^2^	X^2^/df
M-1	0.69	0.29	0.59	0.56	0.27	513.12	9.35
M-2	0.73	0.15	0.67	0.63	0.17	345.56	8.23
M-3A	0.77	0.14	0.73	0.71	0.15	237.69	6.48
M-3B	0.82	0.11	0.81	0.85	0.10	188.12	5.71
M-4	0.92	0.07	0.91	0.90	0.05	171.62	2.56

**Note:** M-1: Public leadership + goal clarity + TMS + PME. M-2: public leadership; goal clarity + TMS + PME. M − 3 A: public leadership; goal clarity + TMS + PME. M − 3 B: public leadership; goal clarity; TMS + PME. M-4: public leadership; goal clarity; TMS; PME.

### 4.2 Descriptive statistics analysis

Table 2 shows the scales' means, standard deviations, correlation, and reliability. The internal consistency of study variables was found within the accepted range. All research variables showed sufficient internal consistency, and correlations fell within the expected pattern (α > 0.70). The correlation between public leadership and goal clarity (*r* = 0.30, *p* < 0.01), PME (*r* = 0.20, *p* < 0.01), and TMS (*r* = 0.38, *p* < 0.01). Furthermore, the correlations between PME and top management support (*r* = 0.24, *p* = 0.01) and goal clarity (*r* = 0.43, *p* = 0.01).

**Table 2:** Mean, SD, and correlation.

Variables	Mean	SD	1	2	3	4	5	6	7
1. Age	2.41	.84	–						
2. Experience	1.69	.78	.023						
3. Education	2.83	.56	.075	−.056					
4. Public Leadership	3.81	.57	.049	.048	−.021	**(0.84)**			
5. Project management Effectiveness	3.35	.44	.012	.047	.039	.205**	**(0.82)**		
6. Top Management Support	3.23	.67	.017	−.050	.014	.385**	.246**	**(0.78)**	
7. Goal Clarity	3.78	.78	.081	.073	.011	.305**	.431**	.334**	**(0.85)**

**Note:** **p < 0.01, Bold values show the reliability value and are presented on a diagonal.

### 4.3 Direct path and the mediating effect

The outcomes of testing hypotheses 1 and 2 using hierarchical linear regression are shown in Table 3. Public leadership showed a substantial positive effect on goal clarity (β = .049, p < 0.001, Model 1) and project management effectiveness (β = 0.032, p < 0.001, Model 2), after controlling for age, years of experience. Gender was represented as a dummy variable with a code of 1 for male and 0 for female. When gender was coded as a dummy variable, the regression results significantly changed. Thus, public leadership can promote employees’ goal clarity and play a key role in project management effectiveness, supporting Hypotheses 1 and 2. Moreover, goal clarity mediates the link between public leadership and project management effectiveness (direct effect = 0.31, SE = 0.032, 95% CI = 0.2648, 0.3952; indirect effect = 0.36, SE = 0.030, 95% CI = 0.2863, 0.3991). Thus, it confirms Hypothesis 3.

**Table 3:** Hierarchical linear regression results.

Variables	Goal Clarity			Project management effectiveness		
	Model 1	Model 2	Model 3	Model 4	Model 5	Model 6
Age	0.04	0.07	0.08	0.09	0.08	0.09
Gender	0.05	0.06	0.07	0.05	0.05	0.07
Education	0.08	0.06	0.08	0.07	0.09	0.06
Service	0.05	0.07	0.06	0.05	0.09	0.06
Public Leadership	.049***			.032***		
**Mediator (Goal Clarity)**					.361***	
**Moderator (TMS)**		.1948***				
**Interaction Effect**						
PL x POS			.1631**			
*R*^*2*^	33.79***	29.84***	24.96**	31.88**	22.97***	27.56***
*F*	0.24	0.25	0.14	0.19	0.21	0.29

**Note:** **. Coefficient is significant at the 0.01 level (2-tailed). ***. Coefficient is significant at the 0.001 level (2-tailed).

## 5 Top management support as a moderator

**Fig. 3:**
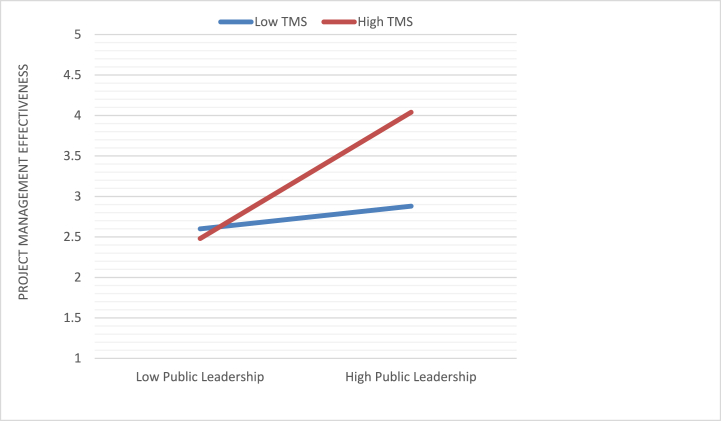
Interaction effect of public leadership and top Management support on goal clarity.

According to Table 3, which supports Hypothesis 4a, top management support significantly moderated the positive association between public leadership and goal clarity (β = .1948, SE = 0.04, p < 0.001, 95% CI = −0.3013, −0.0110). We presented the interaction effect in Fig. 3 to make it simpler to comprehend. Simple slope tests were used to evaluate top management support (i.e., +1and −1 SD from the mean) (Aiken et al., 1991). When top management support is high, our results showed a positive and significant relationship between public leadership and goal clarity (simple slope = 0.97, SE = 0.05, t = 19.4, p < 0.001, 95% CI = 0.8743, 1.0798); as compared to when it is low (simple slope = 0.79, SE = 0.07, t = 11.28, p < 0.001, 95% CI = 0.6340, 0.9465). The results confirm Hypothesis 4a.

### 5.1 Moderation mediation examination

The Hayes [66] PROCESS macro model 7 has been used for the moderation mediation analysis, as shown in Table 4. We performed an integrated moderation mediation analysis of Hypothesis 4b as proposed by Hayes and Rockwood [67]. (Moderator values are the mean and ±1 SD). It was shown that PME, as an outcome variable in a moderated mediated model, was statistically significant when TMS was high (β = 0.2984, SE = 0.0301, 95% CI = 0.2358, 0.3469), as compared to when top management support is low (β = 0.2335, SE = 0.0251, 95% CI = 0.1875, 0.2893). As a result, the moderated mediation index was significant (Index = −0.0456, SE = 0.0184, 95% CI = 0.066, 0.0148). Thus, it confirms Hypothesis 4b.

**Table 4:** Moderated mediated results across levels of TMS.

Moderator	Level	Conditional Indirect Effect	SE	LLCI	ULCI
TMS	Low	.2535	.0251	.1875	.2893
	High	.2984	.0301	.2358	.3469
	Difference	.0449	.005	.0483	.0576

**Note**: Moderator values are the mean and ±1 SD.

## 6 Discussion

Governments around the world implement public plans and projects for socio-economic development. The government of Pakistan delivers public projects for improving socio-economic purposes. The frequent project failure has attracted project management scholars to identify critical factors for project failure and project success. A project leader's behavior is considered one of the most important critical success factors of projects [11,68,69].

This study investigates the relationship between public leadership style, goal clarity, and PME. Current research also focused on public leadership's role in PME through the mediating mechanism of goal clarity. Results revealed that public leadership is significantly associated with project management effectiveness. That is increased experiences and perceptions of public leadership increase project management effectiveness. In other words, public project team members' experiences, a clear goal and perceptions of accountability, rule-following, political loyalty, and network governance leadership behaviors of public officials influence project effectiveness. That is, the perceptions of public project effectiveness vary with the changing perceptions of project officials' public managerial leadership. This relationship is justifiable as researchers have received empirical research evidence that different managerial leadership styles, including transformational leadership [18], humble leadership [3], despotic leadership [70], and servant leadership [71], influence project success and effectiveness.

An earlier study has found that transformational leadership is related to project success in the project management context of Pakistani [72]. Vogel, Reuber [32] argue that public leadership is a public sector-specific transformational leadership behavior/style; therefore, it is highly likely that public leadership is related to PME. The association of public leadership with public project management effectiveness is seen in the light of SLT [73]. The project team members learn the leadership behaviors of accountability, rule-following, political loyalty, a clear goal, and network governance, which direct the team members to stay accountable in their actions to project stakeholders, follow project execution procedures, stay loyal and committed to achieving project outcomes and establish networks with project stakeholders. All these combined are likely to facilitate successful accomplishment projects. According to SLT, the project team members may adopt the same leadership behaviors or other attitudes and behaviors expected to be admired by leadership with public leadership attributes, which facilitate the successful execution of public projects and increase project management effectiveness [18].

Regarding goal clarity and setting, we revealed that a public leader is a significant determinant of goal clarity techniques, verifying earlier study findings [74]. Accumulating the existing literature considers goal clarity an essential component of leader behavior. Furthermore, we found that goal clarity partially mediates the positive impact of public leadership on PME. The current study showed that goal clarity, such as target-setting, task-clarification, human relations, and problem-solving, leads to a highly committed project team and project goal-oriented project team [21].

This research supports the previous claim with a successful goal clarity method. While project managers collaborate and enhance team members' approaches to project goals, activities, and roles, organisational practices and conflict management eventually influence project management effectiveness [20]. It was also noted that goal clarity partially mediates the influence of public leadership on project management effectiveness. This implies that public leadership is partially based on a positive goal clarity mechanism when ensuring the effectiveness of the project. Regarding the moderator role, we introduced top management support as moderator, or more precisely, improved the positive role public of public leadership on goal clarity, including the indirect role on project management effectiveness.

Asian management and leadership mindset may be one reason for this unforeseen outcome [3]. Apparently, in Pakistan's cultural milieu, whenever project managers and subordinates get considerable organisational assistance, they are less amenable to the directors as authoritarian people, making it challenging for elite management to foster goal settings Pakistan's culture milieu, whenever project managers and subordinates get considerable organisational assistance, they are less amenable to the directors as authoritarian people, making challenging for management to foster goal settings [3]. Chull Shin [75] also revealed that organisations are based on autocratic methods and establish roles and goals without involvement from project managers and subordinates. Ahmed, Mohamad [54] asserted that top-level management is not a significant determinant of goal clarity.

According to the prior literature, the positive working behavior of public project leaders can improve goal clarity, enhancing project management effectiveness. Even organisational assistance is only a minor factor. These questions were not widely addressed in the existing research. Therefore, this research advances a gap by confirming how public leadership affects the goal clarity essential for project management effectiveness.

### 6.1 Theoretical implications

The current research has several theoretical contributions to the existing body of knowledge. The first significant theoretical contribution of the study is our empirical finding that public leadership is related to PME. The construct of public leadership is recently conceptualised, and there exist limited empirical studies regarding the outcomes of public leadership. The theorists of the public leadership construct continuously emphasise investigating the role of public leadership on different variables from the public sector context [13,32].

Researchers have noted the role of different mainstream leadership on project success, and researchers and practitioners are continuously exploring different aspects of managerial leadership concerning project management effectiveness. Studies on mainstream leadership approaches and public project effectiveness are also scarce [72]. Our study extends the public leadership constructs to public management by providing an explanatory relationship of public leadership with public project effectiveness. Project goal clarity research has received attention in other disciplines. However, there is insufficient literature regarding goal clarity in public management. There is very little empirical study regarding the relationship between public sector-specific leadership approaches such as public leadership and the construct of goal clarity in public agencies.

The second implication of our finding indicates that goal clarity positively mediates the relationship between public leadership and PME. The vital role of project managers is to create a professional team that performs the tasks, assignments, and knowledge required to achieve the project goals. One implication is conformist goal clarity methods, particularly planned and unintended group-level meetings designed to improve organisational structure, define tasks, and resolve responsibilities and behavioral issues concerning project effectiveness. This ensures that projects will likely be significantly effective if goal clarity elements are used correctly. The past literature has found that an organisation nurtures an environment where goals are transparent and the optimistic project team, thus increasing project effectiveness.

Thirdly, our findings suggest that top management supports efficient resource management, hierarchies, skills, and techniques, increasing the possibility of project management effectiveness and goal clarity. The results would motivate experts to take challenges more enthusiastically to ensure the organisation views its role seriously and that the project manager gets the necessary support during the project. Supportive management contributes to cohesion and creative strategies across the learning atmosphere [55]. Public project managers and adherents can induce time configuration to manage this learning process, mainly when project implementation time is limited. The study reveals that such supportive management energises project leaders and associates to try hard and attain remarkable success, ultimately improving project management effectiveness [76].

### 6.2 Practical implications

This research also has several practical implications. Pakistani public sector developmental projects are approved by governing bodies, planned by planning agencies, and executed by private execution agencies under the supervision and leadership of project heads and their teams in the national socio-economic interest of the general public. All these stakeholders want to see public projects succeed in terms of efficient project completion (within time, cost, and quality standards), providing specific socio-economic impact, organisational benefits, and key stakeholders' satisfaction [77,78], as these Pakistani public sector developmental projects are implemented by teams of professional and board of directors.

We encourage project stakeholders, including project planning and executing agencies and project officials, to focus on project goal clarity as this leads to the successful accomplishment of public sector projects. Efficient project management and project progress require goal clarity that works on identifying ways to set and achieve project goals, team members' involvement in clarifying individual roles, shared responsibilities, and organisational norms, goal clarity, high-quality interpersonal relationships, and improving team members' abilities to identify problems and action plans for resolving that problems [18].

HR departments and project officials may clear the project goal to the team on time to improve team project performance and formation through various training and development programs. Furthermore, our findings suggest that the roles of responsibility, rule-following, political allegiance, and network governance played by project leaders impact the clarity of public project goals and the success of such projects. Therefore, we encourage public project planning and executing agencies to ensure leadership practices of accountability, rule-following, political loyalty, and network governance as project team members learn these leadership behaviors to pursue the same behaviors.

In contrast, executing projects or manifesting in the form of other desirable attitudes and behaviors ultimately influences project goal clarity and project management effectiveness. Top management's support helped public workers have a clear sense of purpose and a strong propensity towards transparency, regulation, and good network governance. Civil servants with these attributes are highly likely to convert public sector projects into successful endeavours, and employees with these attributes form efficient, leading towards project effectiveness.

### 6.3 Limitations and future research directions

This research was effective in several ways. We employed a time-lagged research design in our study to reduce the influence of common method bias. Our study, conducted in Pakistani public sector developmental projects, guaranteed that public leadership, goal clarity, and top management support lead to project effectiveness. When evaluating the data, it is crucial to consider that numerous limitations, such as surveys, may contribute to common method bias. Self-reporting may be suitable for certain research because of its prevalence in the scholarly literature and usefulness as a pattern-matching survey approach [79]. First, the limited size of our sample is a major limitation of our study. The time-lag design led to high dropout rates and a limited sample size. While the results did lend credence to certain hypotheses, a bigger sample size might help generalise those results. Future researchers may verify the relationships by collecting data using the same self-reporting measures at different time intervals to validate further the stability among the relationships of variables of the study. The results of our study are based on constructs of distinct dimensions of the variables of public leadership, goal clarity, top management support, and project management effectiveness. Future researchers may investigate the relationships among different dimensions of the study variables to understand the phenomenon more deeply. Future studies may use a mixed-method approach to verify the research model results. Lastly, we encourage researchers to examine the relationships in other public project management contexts using samples of different respondents.

## 7 Conclusion

Our results confirm that public leadership is crucial as it clarifies the stated, well-defined goals that improve the government and public organisations' operations and tasks. Effective public leadership includes planning, efficiency, openness, and accountability, which are important contributing factors for project management effectiveness. Pakistan's public sector spends a huge amount on developmental projects to improve the socio-economic conditions of societies. Understanding what determines project management effectiveness is essential for Pakistan public sector developmental projects. We examined public leaders, directly and indirectly (via goal clarity), that influence project management effectiveness within Pakistani public sector developmental projects. The study's results reveal that the effectiveness of project management is influenced by goal clarity, which acts as a mediator between public leadership and project management. Moreover, our findings suggest that the relationship between public leadership and goal clarity is moderated by top management support.

## Declarations

### Author contribution statement

Muhammad Zada: conceived and designed the experiments; contributed reagents, materials, analysis tools or data; wrote the paper.

Jawad Khan: conceived and designed the experiments; data collation; analyzed and interpreted the data; wrote the paper section.

Imran Saeed: data collation; analyzed and interpreted the data; wrote the paper section.

Shagufta Zada: data collation; analyzed and interpreted the data; wrote the paper section.

Zhang Yong Jun: Supervision; revision; contributed reagents, materials, analysis tools or data.

### Funding statement

This research did not receive any specific grant from funding agencies in the public, commercial, or not-for-profit sectors.

### Data availability statement

Data will be made available on request

### Declaration of interest’s statement

The authors declare no conflict of interest.
