# Supplementary material for: Linking public leadership with project management effectiveness: Mediating role of goal clarity and moderating role of top management support
Source: Heliyon. 2023 Apr 20;9(5):e15543. doi: 10.1016/j.heliyon.2023.e15543 (PMC10149401; doi:10.1016/j.heliyon.2023.e15543)
Supplement: Multimedia component 1 [file mmc1.doc]

**QUESTIONNARE**

**Demographic Information of respondent**

| Serial Number | |  |
| --- | --- | --- |
| 1 | Age of the Respondent |  |
| 2 | Gender |  |
| 3 | Education |  |
| 4 | work experience |  |

Please tick 🗹 a response for each question.

| *Public leadership* | | | **SA 1** | **A2** | **ND3** | **DA4** | **SD5** |
| --- | --- | --- | --- | --- | --- | --- | --- |
| 1 | My supervisor encourages me and my colleagues to explain our actions to various stakeholders | |  |  |  |  |  |
| 2 | My supervisor encourages us to inform stakeholders of our way of working | |  |  |  |  |  |
| 3 | My supervisor strives to ensure that we openly and honestly share the actions of our organizational unit with others. | |  |  |  |  |  |
| 4 | My supervisor emphasizes to me and my colleagues that it is important to follow the law. | |  |  |  |  |  |
| 5 | My supervisor gives me and my colleagues the means to properly follow governmental rules and regulations. | |  |  |  |  |  |
| 6 | My supervisor ensures that we accurately follow the rules and procedures. | |  |  |  |  |  |
| 7 | My supervisor encourages me and my colleagues not to jeopardize the relationship with political heads, even if that entails risks. | |  |  |  |  |  |
| 8 | My supervisor encourages me and my colleagues to defend political choices, even if we see shortcomings. | |  |  |  |  |  |
| 9 | My supervisor encourages me and my colleagues to invest substantial energy in the development of new contacts. | |  |  |  |  |  |
| 10 | My supervisor motivates me and my colleagues to regularly work together with people from our networks. | |  |  |  |  |  |
| 11 | My supervisor motivates me and my colleagues to develop many contacts with people outside our own department. | |  |  |  |  |  |
| **Top management support (TMS)** | | | **SA 1** | **A2** | **ND3** | **DA4** | **SD5** |
| 1 | | Sufficient incentives were provided by top management (TM) for the implementation of the project |  |  |  |  |  |
| 2 | | The project is viewed as a Strategic activity by TM |  |  |  |  |  |
| 3 | | There was sufficient commitment to the implementation of the project. |  |  |  |  |  |
| 4 | | Sufficient resources were provided to implementation of the project. |  |  |  |  |  |
| 5 | | The general manager of the division actively supported the project. |  |  |  |  |  |
| 6 | | TM identified the implementation of the project as a critical priority |  |  |  |  |  |
| **Goal clarity (GC)** | | | **SA 1** | **A2** | **ND3** | **DA4** | **SD5** |
| 1 | | There were clear and comprehensible goals for this project. |  |  |  |  |  |
| 2 | | The goals and requirements of the customers were clear for this project |  |  |  |  |  |
| 3 | | The goals and requirements of the management were clear for this project. |  |  |  |  |  |
| **Project Management effectiveness (PME)** | | | **SA 1** | **A2** | **ND3** | **DA4** | **SD5** |
| 1 | | There was a detailed plan (including time, schedules, milestone, manpower requirements, etc.) for the completion of the project. |  |  |  |  |  |
| 2 | | Availability of appropriate tools to measure performance should be in place |  |  |  |  |  |
| 3 | | The time limit for the project is clearly stated |  |  |  |  |  |
| 4 | | Project meetings have well-planned agenda |  |  |  |  |  |
| 5 | | In the case of any project delay, your project team has to put some efforts in trying to solve the problem |  |  |  |  |  |
| 6 | | The project has no or minimal technical start-up problems because it was readily accepted by its intended users |  |  |  |  |  |
| 7 | | The project has been completed on time |  |  |  |  |  |
| 8 | | The project has been completed according to the budget allocated |  |  |  |  |  |
| 9 | | The outcomes of the project have usually met the quality standard set by specifications |  |  |  |  |  |
| 10 | | The project has been paid within current year |  |  |  |  |  |

Thanks
